# Inhibitory Effects of High-Hydrostatic-Pressure Processing on Growth and Histamine Formation of Histamine-Forming Bacteria in Yellowfin Tuna Meat during Storage

**DOI:** 10.3390/biology11050702

**Published:** 2022-05-03

**Authors:** Chih-Hsiung Huang, Ching-Yu Hsieh, Yi-Chen Lee, Tsung-Yin Ou, Tien-Hsiang Chang, Shih-Hsiung Lee, Chih-Hua Tseng, Yung-Hsiang Tsai

**Affiliations:** 1 Department of Fisheries Production and Management, National Kaohsiung University of Science and Technology, Kaohsiung 811213, Taiwan; 0628908@nkust.edu.tw; 2 Department of Seafood Science, National Kaohsiung University of Science and Technology, Kaohsiung 811213, Taiwan; chingyu601@gmail.com; 3 Department of Marketing and Distribution Management, National Kaohsiung University of Science and Technology, Kaohsiung 811213, Taiwan; outy@nkust.edu.tw; 4 Department of Intelligent Commerce, National Kaohsiung University of Science and Technology, Kaohsiung 811213, Taiwan; thchang@nkust.edu.tw (T.-H.C.); shlee@nkust.edu.tw (S.-H.L.); 5 Department of Fragrance and Cosmetic Science, Kaohsiung Medical University, Kaohsiung 807378, Taiwan; chihhua@kmu.edu.tw

**Keywords:** non-thermal technology, histamine, tuna, histamine-forming bacteria

## Abstract

**Simple Summary:**

High-hydrostatic-pressure treatment effectively inhibited the growth and histamine formation of histamine-forming bacteria species in tuna meat during storage at low temperatures. Our findings can be used to enhance food safety and decrease the risk of histamine poisoning associated with consumption of tuna meat products.

**Abstract:**

In the research, we evaluated the effects of high-pressure processing (HPP) on the growth and histamine formation of histamine-forming bacteria (HFB) in yellowfin tuna meat during storage. Tuna meat samples inoculated with the individual HFB species *Morganella morganii* and *Photobacterium phosphoreum* were subjected to HPP treatment at 250, 350, 450, and 550 MPa for 5 min, and the changes in bacterial count, total volatile basic nitrogen (TVBN) content, pH, and histamine content during storage at 4 and 15 °C were analyzed. The results indicate that the bacterial counts of the HFB species decreased significantly with increasing pressure, and HFB became undetectable in the samples treated at 450 and 550 MPa. At a storage temperature of 15 °C, the bacterial counts of both HFB species in the control group and samples subjected to HPP treatment at 250 and 350 MPa increased significantly with storage time. The bacterial counts of *M. morganii* in the samples stored at 4 °C decreased, whereas those of *P. phosphoreum* increased gradually owing to its psychrophilic nature. HPP treatment (>250 MPa) inhibited the increases in pH and TVBN content of the samples stored at 15 °C and delayed histamine formation in the samples during storage; these effects were more significant as the pressure during HPP treatment was increased.

## 1. Introduction

Pelagic fish, such as tuna, has a high nutritional value in unsaturated fatty acids and other nutrients that are beneficial to human health [1]. However, these fish are highly perishable due to factors such as near-neutral pH, high water activity, and high protein content and are especially prone to histamine fish poisoning [1]. Histamine is a biogenic amine responsible for histamine fish poisoning, a typically moderate to severe disease characterized by the onset of allergy-like symptoms, also accounting for death, after the consumption of mishandled meat of tuna or related species of the *Scombridae* family [2]. Moreover, they are not only *Scombridae* family species related to histamine fish poisoning but also *Engraulidae*, *Clupidae*, *Coryphenidae*, *Pomatomidae*, and *Scomberesocidae*. Histamine is primarily formed via decarboxylation of free histidine in fish muscle by histidine decarboxylase, which is produced by histamine-forming bacteria (HFB) in aquatic products [3]. In Taiwan, histamine fish poisoning occurs frequently after consumption of yellowfin tuna (*Thunnus albacares*) [3], shortbill spearfish (*Tetrapturus angustirostris*) [4], Japanese Spanish mackerel (*Scomberous niphonius*) [5], and milkfish (*Chanos chanos*) [6]; moreover, consumption of yellowfin tuna is one of the primary causes of histamine fish poisoning [7]. Yellowfin tuna, a highly migratory fish species, which is widely distributed in pelagic ocean waters, is one of the four primary tuna species (albacore, bigeye, skipjack, and yellowfin) and comprises 42% of the total tuna caught by Taiwanese fishing vessels in the Indian Ocean [8]. In Taiwan, yellowfin tuna is primarily sold as frozen (whole fish, loin) or fresh (whole fish, loin) products at fish markets [8]. Moreover, yellowfin tuna is a primary ingredient in canned fish products, and it is consumed as sashimi, sushi, and pan-fried dishes [7].

*Enterobacteriaceae* are the primary species responsible for histamine production in fish products. The most prolific HFB among enteric bacteria are mesophilic bacteria with an optimum growth range from 20 to 45 °C [9]. In particular, *Morganella morganii* has consistently produced high histamine levels in cultures (>1000 ppm) and plays a pivotal role in histamine formation and accumulation during fish meat storage [9,10]. In addition, the marine bacterium *Photobacterium phosphoreum* is a major histamine producer in fish meat stored at temperatures below 15 °C [11]. *P. phosphoreum*, which is a known psychrophilic and halophilic, was the bacterium responsible for the histamine fish poisoning outbreaks in Japan that were caused by consumption of dried sardines [11]. In addition to affecting the quality of fish meat stored at 2–8 °C, *P. phosphoreum* causes the histamine content in fish muscle to increase to hazardous levels (>50 mg/100 g) after prolonged low-temperature storage [9,11].

High-hydrostatic-pressure or high-pressure processing (HPP) is a non-thermal processing method that uses pressures of ≥ 300 MPa to kill spoilage and pathogenic bacteria in food products, thereby assuring consumption safety and prolonging their storage life. HPP promotes better nutrient retention and does not considerably affect aroma compounds, rendering it suitable for processing heat-sensitive foods [12]. HPP has already been broadly applied in the manufacture of fruit and vegetable drinks, meats, and aquatic products [12]. Recently, we inoculated HFB into phosphate-buffered saline (PBS) solutions and fish homogenates and subjected the obtained samples to HPP treatment [13,14]. Our results indicate that the bacterial counts in both media decreased with increased pressure and pressurization time. The D-values of the bacterial strains in the fish homogenates were higher than those in the PBS solutions, indicating that the HFB were more pressure-resistant in fish homogenate. The high-pressure tolerances of different types of HFB strains are considerably different [13,14]. The scanning electron microscopy images of HPP-treated samples revealed that HPP treatment damaged HFB cell walls and membranes. This caused leakage and loss of cytoplasm or other cell contents, which ultimately resulted in bacterial death [13,14]. Furthermore, researchers demonstrated that HPP treatment under 300 and 400 MPa on ground mackerel flesh inoculated with HFB (*M. morganii* and *P. phosphoreum*) could retard HFB growth and histamine formation during storage [15]. Ucak et al. [16] subjected vinegar-pickled Atlantic herring (*Clupea harengus*) inoculated with *Mycobacterium psychrotolerans* to high-pressure (300 and 500 MPa) treatment and reported that the treatment slowed the formation of histamine and other biogenic amines. However, few studies have simulated histamine fish poisoning through contamination of tuna meat surfaces with HFB, followed by investigating the effects of HPP treatment on HFB inactivation and changes in sample histamine content during low-temperature storage. Moreover, the above published studies usually involved two or three different pressure levels (MPa) on ground meats of mackerel or Atlantic herring, studying their impact on histamine formation [15,16]; additionally, the use of HPP treatment for freshness maintenance of fish products through retarding the increases in total volatile basic nitrogen (TVBN) content and pH has been rarely reported.

Therefore, this study evaluated the impact of HPP treatment on the bacterial growth, and histamine and TVBN production of the *M. morganii* and *P. phosphoreum* HFB species in tuna meat samples during storage. In addition, the results were to evaluate the applicability of HPP for lowering the risks of histamine poisoning of tuna flesh.

## 2. Materials and Methods

### 2.1. Tuna Flesh Samples

The fish loins of yellowfin tuna (*Thunnus* *albacares*) were purchased from a local fish supplier in Kaohsiung, Taiwan, and immediately transported over crushed ice to our laboratory. Subsequently, the loin was cut into 3 cm × 3 cm × 2 cm samples (30 g each) on a clean bench, soaked in a 75% ethanol solution for 120 s, rinsed with sterile water, drained, and dried before further experiments.

### 2.2. Preparation and Inoculation of Bacterial Cultures

The *M. morganii* strain used in this study, which was isolated from albacore tuna, was provided by Dr. Cheng-I Wei at University of Maryland, USA [10], and the *P. phosphoreum* strain (BCRC 13804) was purchased from the Bioresource Collection and Research Center (Hsinchu, Taiwan). Stock cultures of *M. morganii* and *P. phosphoreum* were stored on trypticase soy agar (TSA; BD Difco, Sparks, MD, USA) and marine agar (BD Difco, Sparks, MD, USA), respectively, at 4 °C. *M. morganii* was inoculated into 100 mL of trypticase soy broth (TSB; BD Difco, Sparks, MD, USA) using a platinum inoculating loop and incubated at 35 °C for 24 h. *P. phosphoreum* was inoculated into TSB containing a 2% sodium chloride solution and incubated at 20 °C for 48 h. Each culture solution was centrifuged at 6000× *g* for 8 min at refrigerated temperature, and the cellular precipitates were washed with PBS (Sigma-Aldrich, Corp, St Louis, MO, USA). Subsequently, the cellular precipitates were resuspended using PBS, and cell suspension was modified to a concentration of 10^8^ CFU/mL. Next, 300 μL of each bacterial suspension was dropped and spread evenly on the surface of a 30 g tuna meat sample, and then the samples were allowed to rest for 5 min to reach an approximate bacterial concentration of up to 10^6^ CFU/g after absorption. The actual number of bacteria adsorbed on the sample is determined in Section 2.5. The inoculated samples were individually vacuum packed for subsequent HPP treatment.

### 2.3. High-Pressure Processing

HPP treatment was carried out using an HPP processing system with a capacity of 6.2 L, maximum operating pressure of 600 MPa, pressure ramp rate of approximately 45 MPa/s, and depressurization time of <10 s (KeFa High-Pressure Technology, Baotou, Inner Mongolia, China). We used 20 °C water as the pressure-transmitting medium. Samples were subjected to HPP treatment at 250, 350, 450, and 550 MPa for 5 min (excluding the pressure ramping and depressurization times). A control group was also prepared using tuna meat samples inoculated with HFB but not subjected to HPP treatment.

### 2.4. Sample Storage Conditions

A storage experiment was performed by storing the HPP-treated samples at 4 and 15 °C. Storage temperatures, which were selected to simulate refrigeration conditions (4 °C) and temperature abuse (15 °C) in mishandled fish meat, have been previously used in a study on the storage life of marine fish [17]. In addition, 15 °C is the temperature that simulates the traditional market when the fish is sold on crushed ice. The control and inoculated tuna meat samples were stored at 4 °C for 15 days (d), and specimens were collected every 3 d. In addition, the control and inoculated tuna meat samples were stored at 15 °C for 7.5 d, and specimens were collected every 1.5 d. According to the results of the preliminary experiment, it was estimated that the growth rate of HFB bacterial number when stored at 15 °C is more than twice that of when stored at 4 °C. Therefore, samples were set to be sampled every 3 d when stored at 4 °C and every 1.5 d when stored at 15 °C. Three individual tuna meat samples from control and each HPP treatment group were gathered at each sampling time and used to determine changes in HFB count, pH, and histamine and TVBN contents.

### 2.5. Histamine-Forming Bacteria Counts

Tuna meat samples (10 g) from the HPP treatment and control groups were added to sterile saline solution (90 mL), followed by homogenization at 3000 rpm for 120 s using a sterilized homogenizer (Omni International, Waterbury, CT, USA) and 10-fold serial dilution using sterile saline. The 0.1 mL of each diluted homogenate was spread on TSA containing 0.5% NaCl (for *M. morganii*) or 2% NaCl (for *P. phosphoreum*) in duplicate. Thereafter, the *M. morganii*- and *P. phosphoreum*-cultured plates were incubated at 35 °C for 24 h and at 20 °C for 48 h, respectively, followed by bacterial counting. All counts are reported as means ± standard deviations of the data collected from three independent experiments.

### 2.6. pH and TVBN Content

pH measurements: tuna meat samples (5 g) were added to deionized water (20 mL), followed by grinding at 2500 rpm for 2 min using a blender (Polytron PT-MR 6100, Kinematica, AG, Littau, Switzerland). The pH values of the mixtures were measured using a PB-10 pH meter (Satorius, Gottingen, Germany).

TVBN content measurements: the TVBN contents of the tuna meat samples were measured using the Conway microdiffusion method [18]. Tuna meat samples (5 g) and a 6% trichloroacetic acid (TCA) (Sigma-Aldrich, Corp, St Louis, MO, USA) solution (20 mL) were mixed in a 50 mL centrifuge tube and homogenized at 5000 rpm for 1.5 min using a homogenizer (Polytron, Model PT3000, Kinematica, AG, Littau, Switzerland). The homogenates were then centrifuged at 3500× *g* and 4 °C for 8 min, followed by filtration through Advanec No. 1 filter paper. These steps were repeated twice, and the final filtrates were collected and topped to 50 mL using a 6% TCA solution to obtain the TCA extracts of the tuna meat samples. Next, each TCA extract (1 mL) and a saturated K_2_CO_3_ (Sigma-Aldrich, Corp, St Louis, MO, USA) solution (1.0 mL) were mixed in the outer ring of a Conway dish, and a boric acid solution (1 mL) was added to the inner ring. After covering the lid, the Conway dishes were then set in an incubator at 45 °C and allowed to react for 60 min. Subsequently, the 0.02 N HCl was titrated into the boric acid absorber in the inner ring of each dish to reach the titration endpoint, and the determined TVBN contents were expressed as milligrams/100 g of tuna meat sample.

### 2.7. Histamine Content

According to the analytical method of Chen et al. [4], 1.0 mL of a histamine standard (Sigma-Aldrich, Corp, St Louis, MO, USA) and 1.0 mL of each TCA extract were added to screw-cap glass tubes. After the addition of 200 μL of 2 N NaOH and 300 μL of a saturated sodium bicarbonate solution to each test tube, 2 mL of acetone solution containing dansyl chloride was added; the test tube contents were uniformly mixed by shaking and then placed to react at 40 °C for 45 min. Upon reaction completion, 100 μL of aqueous ammonia was added to each test tube at 25 °C over 30 min to remove the residual dansyl chloride. Thereafter, the mixtures were brought to final volumes of 5 mL with acetonitrile. Subsequently, the mixtures were centrifuged at 3000 rpm for 4 min. After filtering the supernatant with nylon membrane filters of 0.45 μm, 20 μL of each filtrate was injected into an HPLC system (Hitachi, Ibaraki, Japan) featuring a LiChrospher 100 (5 μm) RP-18 reverse chromatography column (125 mm × 4 mm i.d., E. Merck, Darmstadt, Country) to analyze its histamine content. The HPLC operating conditions were as follows: mobile phase A: acetonitrile, mobile phase B: water; gradient elution program: initial A:B = 50%:50%, A increased to 90% after 19 min, A decreased to 50% at 20 min and held for 10 min (total analysis time: 30 min); flow rate: 1.1 mL/min; column holding temperature: 40 °C; ultraviolet wavelength: 254 nm [4].

### 2.8. Statistical Analysis

The differences in HFB counts, pH levels, and TVBN and histamine contents between tuna meat samples subjected to different HPP treatments were analyzed. Statistical analysis was performed using one-way analysis of variance and the SPSS 12.0 software (St. Armonk, New York, USA). The data are reported as means ± standard deviations of three measurements, and the results were compared using the Tukey test. Differences were considered to be statistically significant at *p* < 0.05.

## 3. Results and Discussion

### 3.1. Changes in Bacterial Counts of the HPP-Treated HFB-Inoculated Tuna Meat Samples during Storage

Table 1 summarizes the bacterial counts of *M. morganii*-inoculated tuna meat samples (Mm-inoculated samples) and *P. phosphoreum*-inoculated tuna meat samples (Pp-inoculated samples) after HPP treatment at 250, 350, 450, and 550 MPa for 5 min. Upon increasing the applied pressure, the *M. morganii* and *P. phosphoreum* counts of the tuna meat samples decreased significantly. The bacterial counts of the Mm-inoculated samples significantly decreased from 6.27 log CFU/g for the control group to <2.0 log CFU/g for the HPP treatment at 550 MPa group (*p* < 0.05). Similarly, the bacterial counts of the Pp-inoculated samples decreased from 5.93 log CFU/g for the control group to <2.0 log CFU/g for the HPP treatment at 550 MPa group (*p* < 0.05). These results indicate that under identical HPP treatment conditions (250 and 350 MPa), the bacterial counts of the Pp-inoculated samples decreased more than those of the Mm-inoculated samples. This was ascribed to the higher tolerance of *M. morganii* to high pressure, as demonstrated by the higher D-value of *M. morganii* compared with that of *P. phosphoreum* under identical pressure conditions [13].

**Figure 1:**
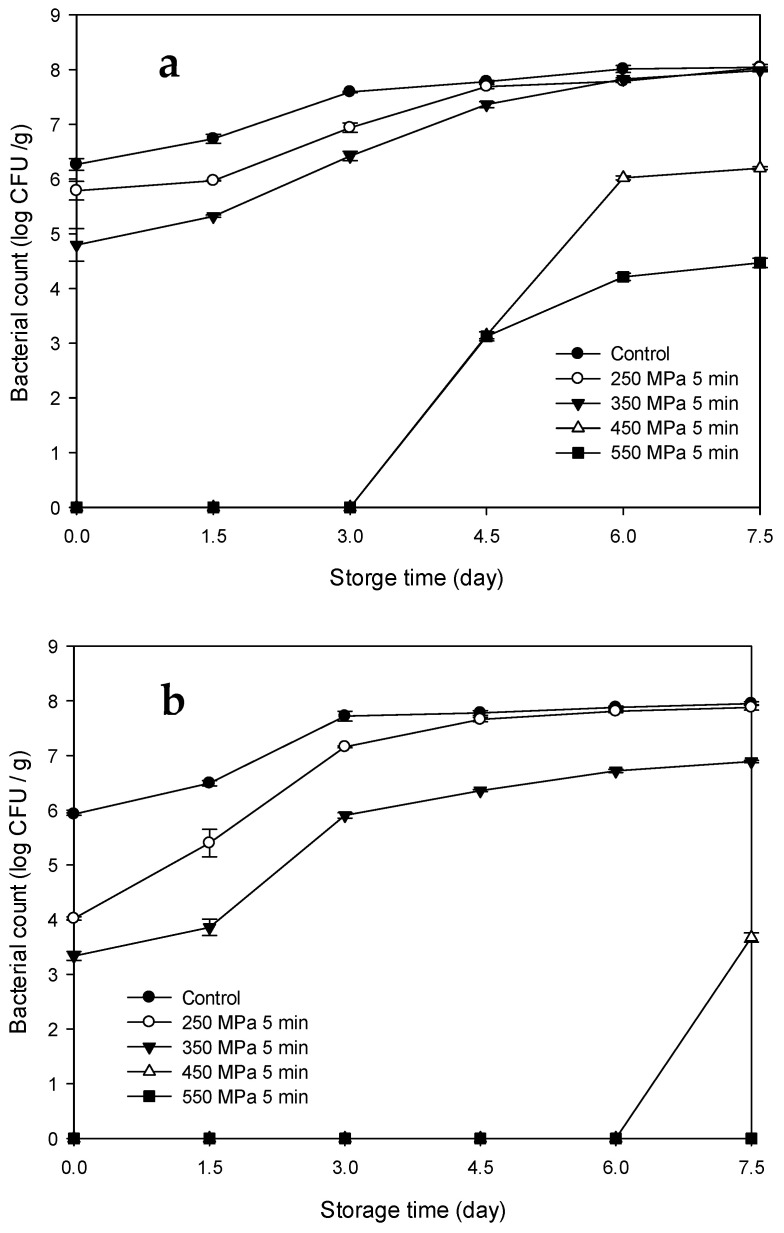
Changes in bacterial counts of tuna meat samples inoculated with (**a**) *Morganella morganii* and (**b**) *Photobacterium phosphoreum* after high-pressure processing treatment at 250, 350, 450, and 550 MPa for 5 min during storage at 15 °C.

The changes in the bacterial counts of the Mm-inoculated samples and Pp-inoculated samples stored at 15 °C after HPP treatment are illustrated in Figure 1a,b, respectively. The bacterial counts of the Mm-inoculated samples subjected to HPP treatment at 250 and 350 MPa were obviously lower than those of the control samples over the first 3 and 4.5 d, respectively (*p* < 0.05); however, the differences in bacterial counts became negligible thereafter. By contrast, the bacterial counts of the Mm-inoculated samples subjected to HPP treatment at 450 and 550 MPa were significantly lower than those of the control and Mm-inoculated samples subjected to HPP treatment at 250 and 350 MPa throughout the entire storage period (*p* < 0.05). The bacterial counts of the Pp-inoculated samples subjected to HPP treatment at 250 MPa were obviously lower than those of the control samples over the first 3 d (*p* < 0.05); however, the differences in bacterial counts became negligible thereafter (Figure 1b). Furthermore, the bacterial counts of the Pp-inoculated samples subjected to HPP treatment at 350 MPa were significantly lower than those of the control and Pp-inoculated samples subjected to HPP treatment at 250 MPa over the entire storage period (*p* < 0.05). Overall, the bacterial counts of the Pp-inoculated samples subjected to HPP treatment at 450 and 550 MPa did not increase throughout the entire storage period; however, the bacterial count of the Pp-inoculated samples subjected to HPP treatment at 450 MPa increased on day 7.5. These findings indicated that higher pressures were more effective in delaying *P. phosphoreum* growth than lower pressures.

**Figure 2:**
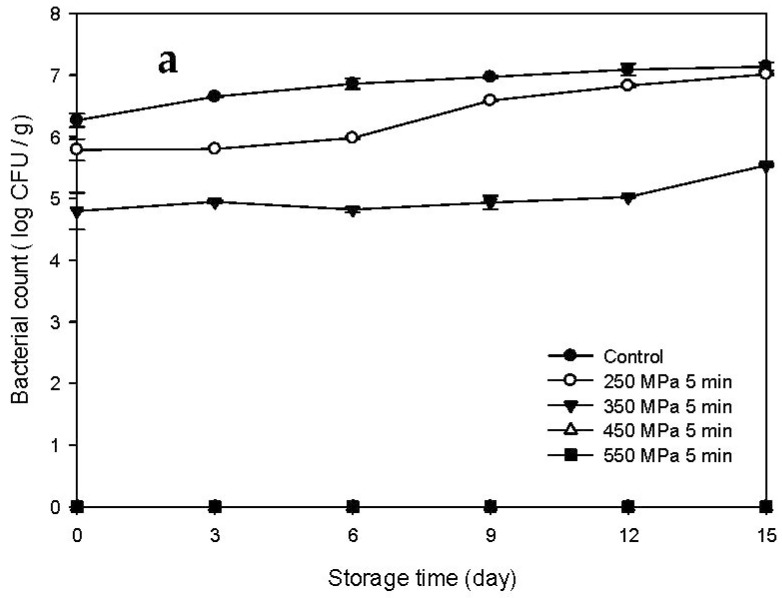

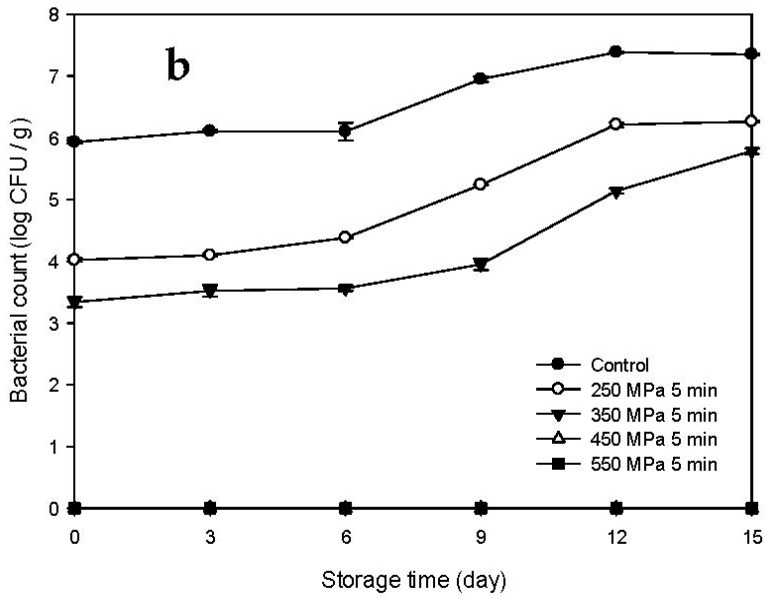
Changes in bacterial counts of tuna meat samples inoculated with (**a**) *Morganella morganii* and (**b**) *Photobacterium phosphoreum* after high-pressure processing treatment at 250, 350, 450, and 550 MPa for 5 min during storage at 4 °C.

The changes in the bacterial counts of the Mm-inoculated samples and Pp-inoculated samples stored at 4 °C after HPP treatment are illustrated in Figure 2a,b, respectively. The bacterial counts of the Mm-inoculated samples increased gradually with increasing storage time for the control and HPP-treated samples at 250 and 350 MPa and reached 7.27, 7.10, and 5.60 log CFU/g, respectively, by the end of the storage period (day 15); that is, the bacterial counts increased by 1.0, 1.31, and 0.8 log CFU/g, respectively. The bacterial counts of the Mm-inoculated samples subjected to HPP treatment at 250 MPa were significantly lower than those of the control samples over the first 12 d (*p* < 0.05); however, the differences in bacterial counts became negligible thereafter. Furthermore, the bacterial counts of the Mm-inoculated samples subjected to HPP treatment at 350 MPa were significantly lower than those of the control and Mm-inoculated samples subjected to HPP treatment at 250 MPa throughout the entire storage period (*p* < 0.05). Furthermore, the bacterial counts of the Mm-inoculated samples subjected to HPP treatment at higher pressures (450 and 550 MPa) were undetectable over the entire storage period. The bacterial counts of the Pp-inoculated control and HPP-treated samples at 250 and 350 MPa increased gradually with storage time and reached 7.35, 6.26, and 5.79 log CFU/g, respectively, by the end of the storage period (day 15); that is, the bacterial counts increased by 1.42, 2.24, and 2.45 log CFU/g, respectively (Figure 2b). The bacterial counts of the Pp-inoculated samples subjected to HPP treatment at 250 and 350 MPa were significantly lower than those of the control samples over the entire storage period (*p* < 0.05), whereas those of the Pp-inoculated samples subjected to HPP treatment at 450 and 550 MPa were undetectable. Therefore, under identical HPP treatment and storage temperature conditions (4 °C), the bacterial counts of the Pp-inoculated samples increased more than those of the Mm-inoculated samples. This was attributed to the psychrophilic nature of *P. phosphoreum*, which enabled the residual bacteria after HPP treatment to continue to grow despite the low storage temperature (4 °C).

These results demonstrate that higher pressures were more effective at delaying the growth of the *M. morganii* and *P. phosphoreum* HFB species. During storage at 4 °C, the *P. phosphoreum* counts of the control and lower-pressure (250 and 350 MPa) HPP-treated samples increased with storage time, indicating that the growth of the residual *P. phosphoreum* was not inhibited by low temperature. Therefore, a continuous increase in *P. phosphoreum* count can occur in contaminated fish meat during refrigeration. In contrast, *P. phosphoreum* growth can be completely inhibited via high-pressure (≥450 MPa) processing followed by storage at 4 °C. In addition, similar results were also reported by Kani et al. [11] that *P. phosphoreum* YS4-7 isolated from dried sardine implicated in histamine fish poisoning continued to grow when cultured at 4 °C for 14 d. In contrast, the growth of *M. morganii* JCM 1672 was inhibited during 14 d when refrigerated at 4 °C [11].

### 3.2. pH Changes of the HPP-Treated and HFB-Inoculated Tuna Meat Samples during Storage

**Figure 3:**
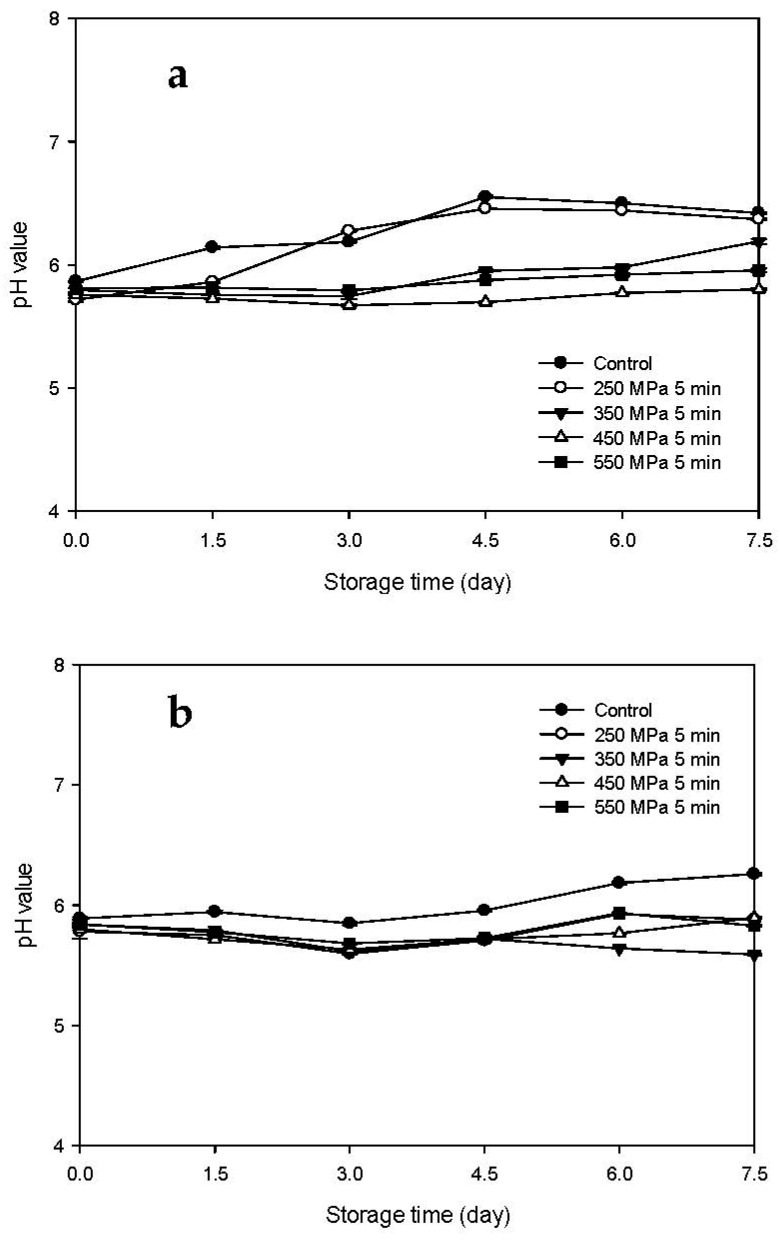
Changes in pH of tuna meat samples inoculated with (**a**) *Morganella morganii* and (**b**) *Photobacterium phosphoreum* after high-pressure processing treatment at 250, 350, 450, and 550 MPa for 5 min during storage at 15 °C.

Changes in pH of the Mm-inoculated samples and Pp-inoculated samples stored at 15 °C after HPP treatment are presented in Figure 3a,b, respectively. The pH values of the Mm-inoculated control and HPP-treated samples at 250 MPa increased gradually with storage time and were significantly higher than those of the HPP-treated samples at 350 and 450 MPa (*p* < 0.05) (Figure 3a). However, the pH values of the 550 MPa HPP treated samples did not change significantly during storage. In addition, the pH values of the Pp-inoculated control samples increased slowly with increasing storage time and were significantly higher than those of the high-pressure-treated samples (*p* < 0.05), while those of all HPP treated samples (250–550 MPa) did not change significantly during storage. As shown in the results from Mm-inoculated samples and Pp-inoculated samples, the increase in pH during storage can be caused by the production of alkaline compounds, including ammonia and amines, during the bacterial growth process [19].

**Figure 4:**
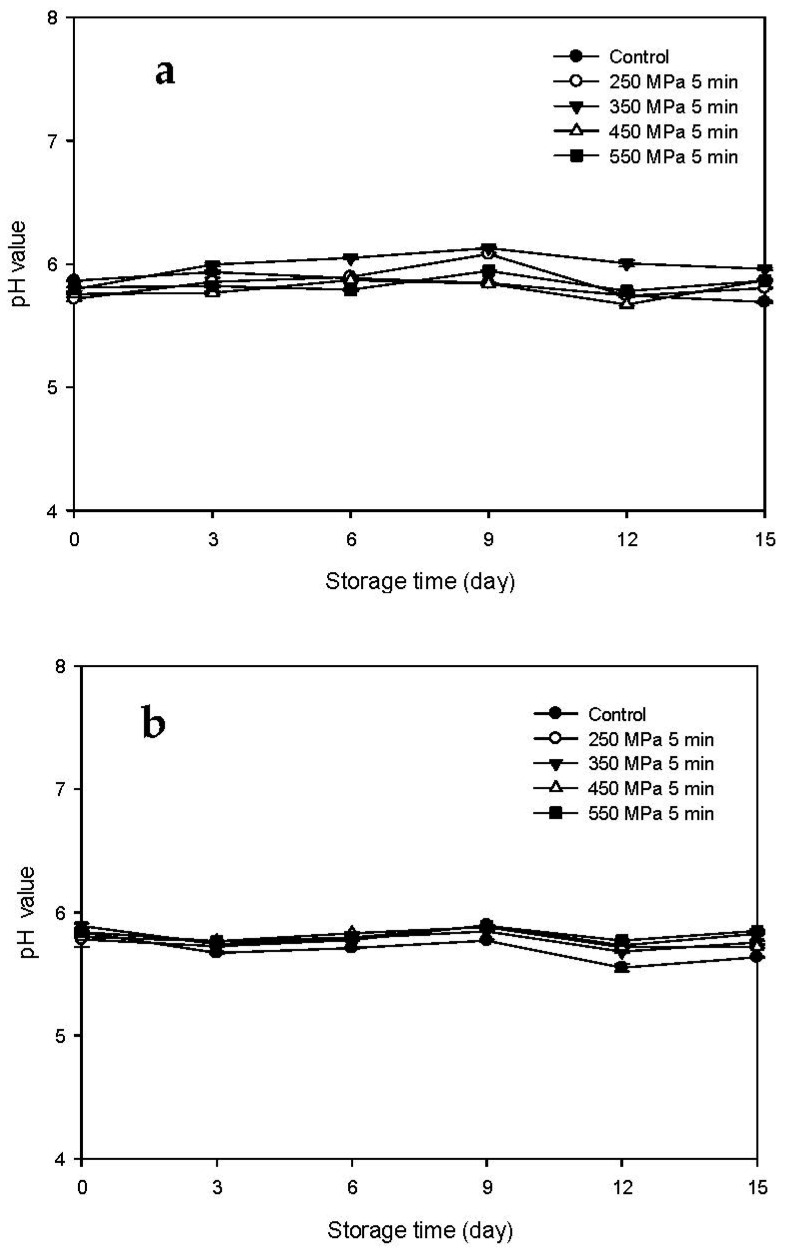
Changes in pH of tuna meat samples inoculated with (**a**) *Morganella morganii* and (**b**) *Photobacterium phosphoreum* after high-pressure processing treatment at 250, 350, 450, and 550 MPa for 5 min during storage at 4 °C.

The pH changes of the Mm-inoculated samples and Pp-inoculated samples stored at 4 °C after HPP treatment are presented in Figure 4a,b, respectively. The pH values of the control and HPP-treated samples did not change significantly with storage time. The pH values of the Mm-inoculated samples and Pp-inoculated samples ranged between 5.76 and 6.13 (Figure 4a) and between 5.55 and 5.89 (Figure 4b), respectively, indicating that storage at 4 °C effectively delayed the increase in pH for all the samples regardless of HPP treatment. In addition, similar results were also presented by Kung et al. [20] who found that the pH value of milkfish meat inoculated with histamine-producing bacteria was not changed much when stored at 4 °C for 12 days. This may be attributable to the fact that refrigerated low temperature inhibits the activity of alkali-producing and ammonia-producing enzymes [20].

### 3.3. TVBN Changes of the HPP-Treated HFB-Inoculated Tuna Meat Samples during Storage

**Figure 5:**
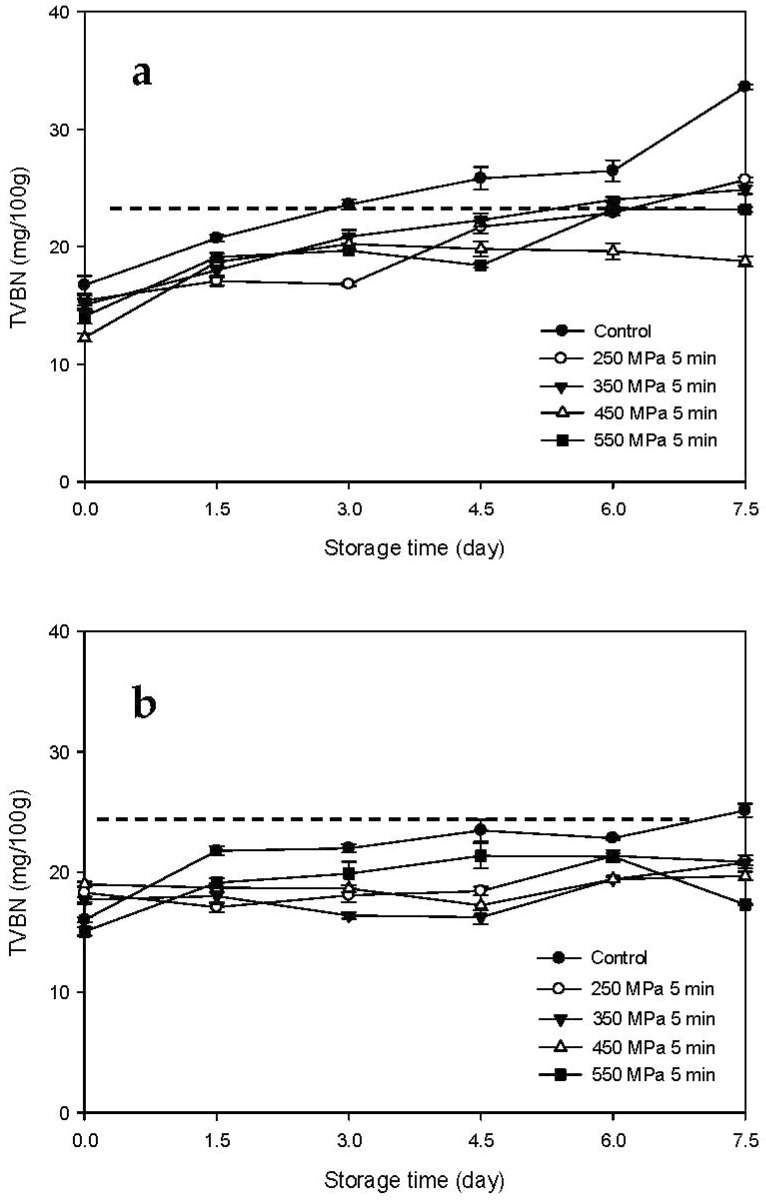
Changes in total volatile basic nitrogen (TVBN) contents of tuna meat samples inoculated with (**a**) *Morganella morganii* and (**b**) *Photobacterium phosphoreum* after high-pressure processing treatment at 250, 350, 450, and 550 MPa for 5 min during storage at 15 °C; dash line indicates the sanitation standard for fish products as 25 mg/100 g of TVBN.

The changes in the TVBN contents of the Mm-inoculated samples and Pp-inoculated samples stored at 15 °C after HPP treatment are illustrated in Figure 5a,b, respectively. The TVBN contents of the Mm-inoculated control samples increased gradually with increasing storage time. By day 4.5, the TVBN contents of the Mm-inoculated control samples (25.83 mg/100 g) exceeded the tolerance limit stated in the Sanitation Standard for Fish Products promulgated by the Taiwan Food and Drug Administration (25 mg/100 g) [21]. The TVBN contents of the Mm-inoculated HPP-treated samples also increased gradually with increasing storage time. The TVBN contents of the Mm-inoculated samples subjected to HPP treatment at 250 MPa exceeded the tolerance limit only at the end of the storage period (day 7.5, 25.69 mg/100 g). However, the TVBN contents of all the other *Mm*-inoculated samples subjected to HPP treatment were lower than 25 mg/100 g over the entire storage period (Figure 5a). The TVBN contents of the Pp-inoculated samples increased gradually with increased storage time and exceeded the tolerance limit at the end of the storage time for the control samples (day 7.5, 25.13 mg/100 g). In contrast, the TVBN contents of the HPP-treated samples remained lower than 25 mg/100 g throughout the storage time (Figure 5b).

**Figure 6:**
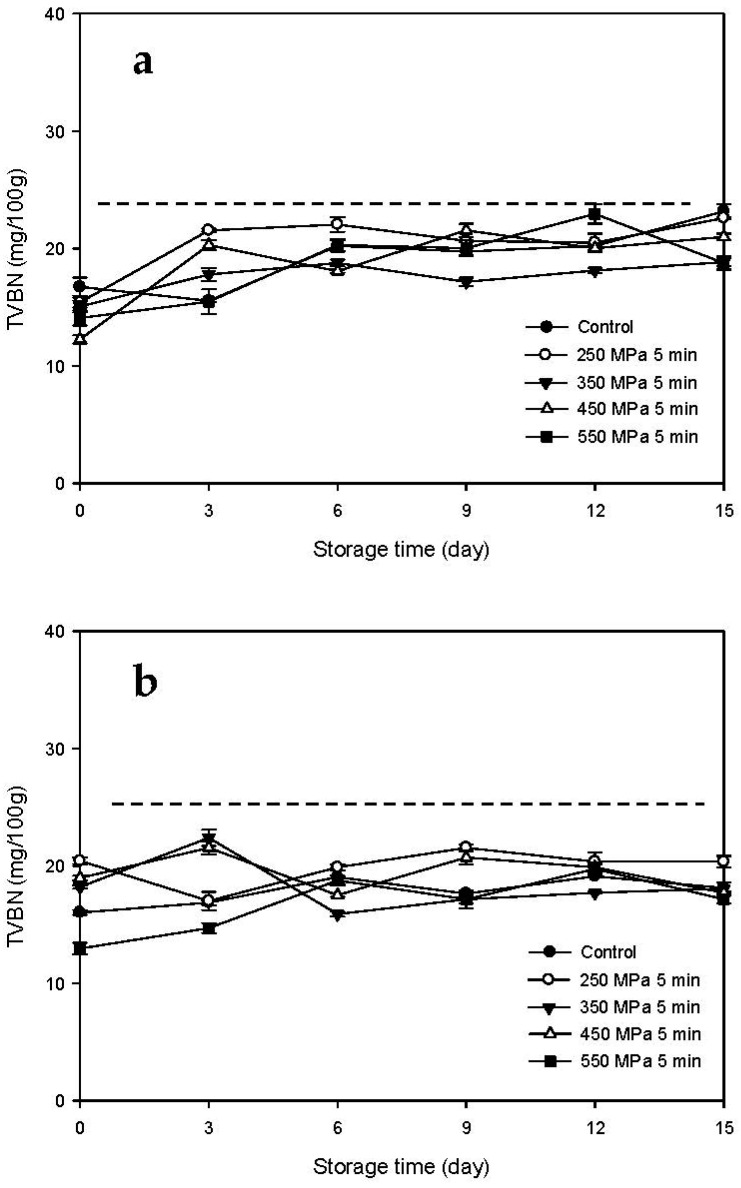
Changes in total volatile basic nitrogen (TVBN) contents of tuna meat samples inoculated with (**a**) *Morganella morganii* and (**b**) *Photobacterium phosphoreum* after high-pressure processing treatment at 250, 350, 450, and 550 MPa for 5 min during storage at 4 °C; dash line indicates the sanitation standard for fish products as 25 mg/100 g of TVBN.

The changes in the TVBN contents of the Mm-inoculated samples and Pp-inoculated samples stored at 4 °C after HPP treatment are illustrated in Figure 6a,b, respectively. The TVBN contents of the two types of samples did not change significantly throughout the storage period regardless of the type of HFB inoculated or HPP processing pressure, and all the TVBN contents were lower than the tolerance limit of 25 mg/100 g. These results indicate that storage at 4 °C was sufficient for delaying the increase in TVBN contents and maintaining the freshness of HFB-contaminated tuna meat samples.

As shown in this study described above, under 15 °C storage, the pre-treated HPP at >250 MPa could delay the increase in the TVBN contents of HFB-contaminated fish meat samples compared to the non-HPP treated sample. However, low-temperature (4 °C) storage can delay significantly the increase in TVBN content of fish meat as compared to higher-temperature (15 °C) storage, regardless of HPP treatment. In addition, our results are in agreement with a previous study by Lee et al. [22], in which the TVBN value of tuna meat inoculated with HFB at 4 °C was significantly lower than that at 15 °C. This may be attributed to the fact that the low temperature of refrigeration inhibits the growth of bacteria and the activity of ammonia-producing enzymes [22].

### 3.4. Changes in the Histamine Contents of the HPP-Treated HFB-Inoculated Tuna Meat Samples during Storage

**Figure 7:**
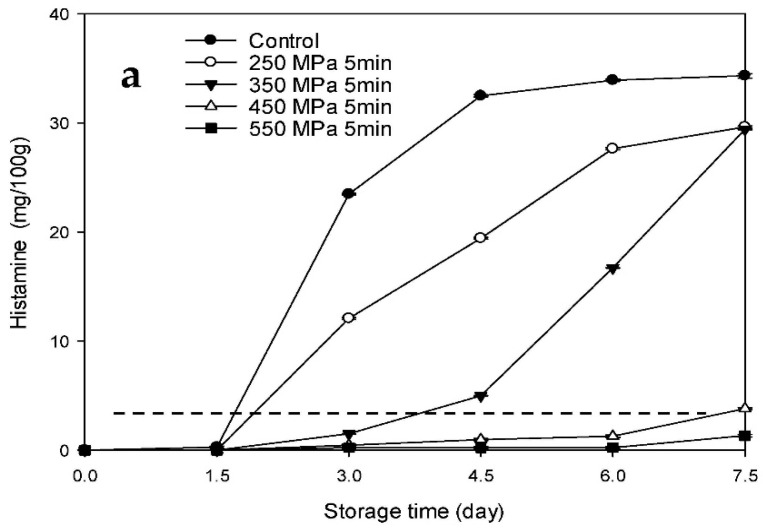

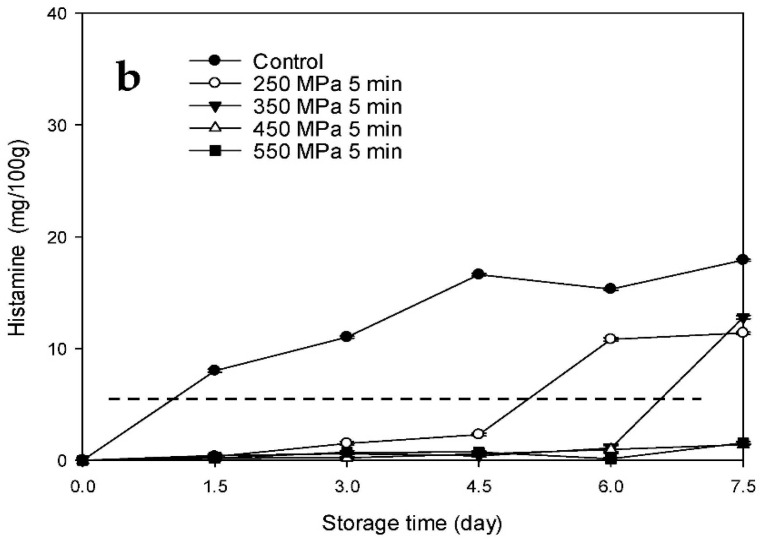
Changes in histamine contents of tuna meat samples inoculated with (**a**) *Morganella morganii* and (**b**) *Photobacterium phosphoreum* after high-pressure processing treatment at 250, 350, 450, and 550 MPa for 5 min during storage at 15 °C; dash line indicates the USFDA tolerance limit of 5.0 mg/100 g of histamine.

The changes in the histamine level in the Mm-inoculated samples and Pp-inoculated samples stored at 15 °C after HPP treatment are presented in Figure 7a,b, respectively. The histamine contents of the Mm-inoculated control and HPP-treated samples at 250 MPa increased rapidly after 1.5 d of storage (Figure 7a), whereas the histamine contents of the HPP-treated samples at 350 MPa increased gradually over the first 4.5 d, followed by a steep increase. The histamine contents of the control and HPP-treated samples at 250 and 350 MPa were 34.8, 28.9, and 28.2 mg/100 g, respectively, at the end of the storage time (day 15). However, the histamine contents of the HPP-treated samples at 250 and 350 MPa were obviously lower than those of the control samples over the same storage period (*p* < 0.05). The histamine levels of the control and HPP-treated samples at 250 MPa on day 3 (23.5 and 12.1 mg/100 g, respectively) and those of the HPP-treated samples at 350 MPa group on day 6 (17.0 mg/100 g) exceeded the histamine content tolerance limit established by the United States Food and Drug Administration (USFDA) (5 mg/100 g; Figure 7a) [23]. By contrast, the histamine contents of the HPP-treated samples at 450 and 550 MPa did not exceed 5 mg/100 g throughout the storage time. For the Pp-inoculated samples, the histamine contents of the samples in the control group increased rapidly over the entire storage period, whereas those of the HPP-treated samples at 250 and 350 MPa increased gradually. Moreover, the histamine contents of the HPP-treated samples at 250 and 350 MPa were obviously lower than those of the control samples at any storage time (*p* < 0.05) *(*Figure 7b). The histamine levels of the control and HPP-treated samples at 250 and 350 MPa exceeded the USFDA tolerance limit of 5 mg/100 g on days 1.5, 6, and 7.5 (8.0, 10.5, and 12.08 mg/100 g, respectively) (Figure 7b). By contrast, the histamine contents of the HPP-treated samples at 450 and 550 MPa did not exceed the tolerance limit throughout the storage time.

**Figure 8:**
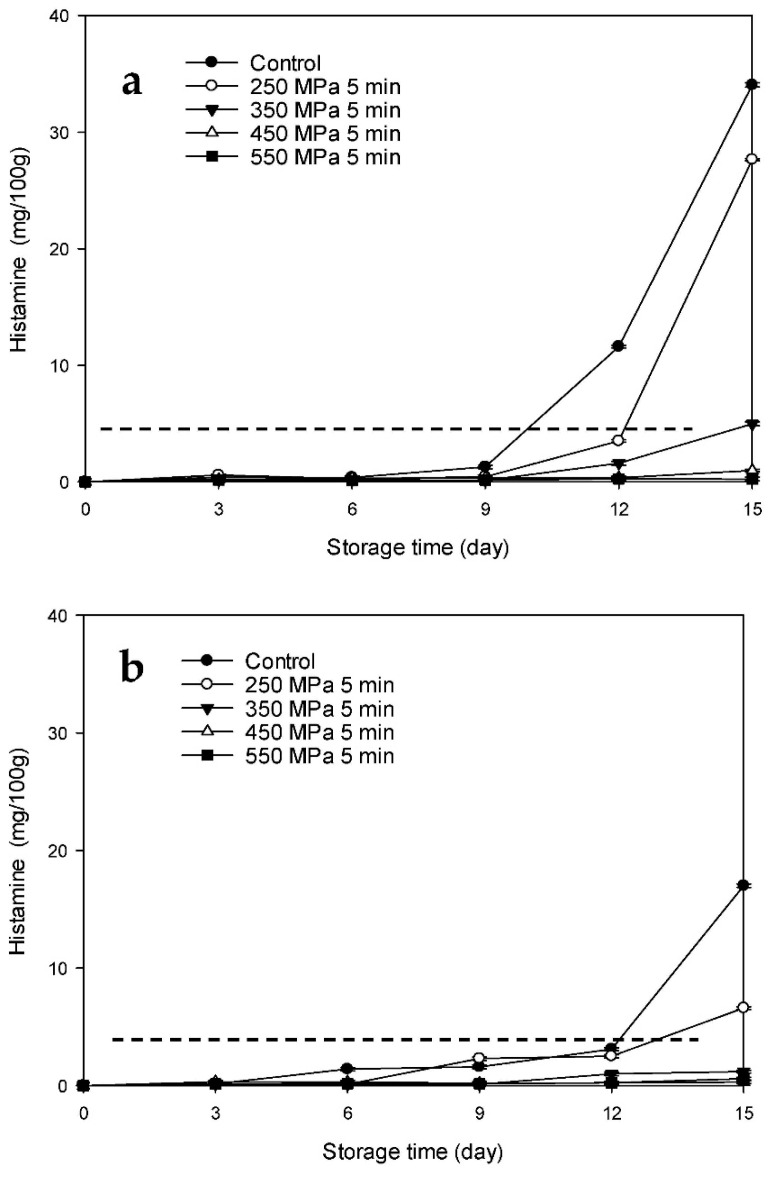
Changes in histamine contents of tuna meat samples inoculated with (**a**) *Morganella morganii* and (**b**) *Photobacterium phosphoreum* after high-pressure processing treatment at 250, 350, 450, and 550 MPa for 5 min during storage at 4 °C; dash line indicates the USFDA tolerance limit of 5.0 mg/100 g of histamine.

The changes in the histamine contents of the Mm-inoculated samples and Pp-inoculated samples stored at 4 °C after HPP treatment are presented in Figure 8a,b, respectively. The histamine contents of the Mm-inoculated control and HPP-treated samples at 250 MPa increased rapidly after 9 d of storage and reached 33.0 and 27.6 mg/100 g, respectively, at the end of the storage time (day 15) (Figure 8a). Histamine contents of the control samples after 12 d of storage and those of the HPP-treated samples at 250 MPa after 15 d of storage (11.6 and 27.6 mg/100 g, respectively) exceeded the USFDA tolerance limit of 5 mg/100 g. By contrast, the histamine contents of the HPP-treated samples at 350, 450, and 550 MPa were lower than 5 mg/100 g throughout the entire storage period. The histamine contents of the Pp-inoculated control and HPP-treated samples at 250 MPa increased rapidly after 12 d of storage and reached 17.0 and 6.6 mg/100 g, respectively, by the end of the storage time (day 15). These levels exceeded the USFDA tolerance limit (Figure 8b). By contrast, the histamine contents of the Pp-inoculated samples subjected to HPP treatment at 350, 450, and 550 MPa did not exceed 5 mg/100 g over the entire storage time. These results indicate that HPP processing (>250 MPa) can retard the increase in histamine content of HFB-contaminated tuna meat samples during storage at low temperatures (4 and 15 °C), and the delay was more significant at higher pressures. Low storage temperatures (e.g., 4 °C) can further delay the increase in histamine content.

Kim et al. [15] reported that the histamine-forming ability of *M. morganii* is stronger than that of *P. phosphoreum*. In addition, histamine formation in fish meat is slow at 4 °C, which demonstrated that refrigerated storage can delay the increase in the histamine level of fish meat. The inhibitory effect of the HPP treatment on histamine formation in HFB-inoculated tuna meat was ascribed to the high-pressure-induced delay in the early growth phase of the bacterial growth cycles of *M. morganii* and *P. phosphoreum* prolonging the lag phase, as well as possibly inhibiting histidine decarboxylase synthesis in bacterial cells [15]. Patterson [24] and Smelt [25] reported that high pressure can affect gene and protein expression, disrupting the enzyme-mediated steps of DNA replication and transcription. In other words, HPP affected the genetic mechanisms of HFB, thereby inhibiting histidine decarboxylase synthesis by bacteria and ultimately decreasing the histamine content of fish meat.

Although HPP treatment had a significant effect on retarding the growth of HFB and histamine production in contaminated tuna meat, the HPP technique has some drawbacks on the quality of tuna meat, including whiter and opaque appearance and color, light-cooked sensory quality, and harder textures [7]. Therefore, future research is needed to overcome or solve the adverse effects of high-pressure processing on fish meat.

## 4. Conclusions

HPP may have some adverse influence on the color, appearance, and texture of tuna meat. However, this study exhibited that HPP processing (>250 MPa) effectively delayed the increases in bacterial count and TVBN and histamine contents of *M. morganii*- and *P. phosphoreum*-contaminated tuna meat samples stored at 4 and 15 °C. Higher-pressure (>450 MPa) treatment and lower-temperature (4 °C) storage enabled the complete inhibition of HFB growth and caused a significant delay in histamine formation (i.e., the histamine contents of the analyzed samples were lower than the USFDA tolerance limit of 5 mg/100 g throughout the storage time). Therefore, we believe that HPP treatment at a pressure of at least 350 MPa for 5 min can effectively inhibit HFB growth in tuna meat and reduce the risk of histamine fish poisoning. Our results suggest that HPP can serve as a novel alternative processing method for delaying microbial growth and diminishing quality loss in fish meat.

## Figures and Tables

**Table 1:** Bacterial counts of tuna meat samples inoculated with *Morganella morganii* and *Photobacterium phosphoreum* after high-pressure processing treatment at 250, 350, 450, and 550 MPa for 5 min.

Treatment	*Morganella morganii*		*Photobacterium phosphoreum*	
	Bacterial Counts	Reduction Counts	Bacterial Counts	Reduction Counts
Control	6.27 ± 0.11 *^a^	0	5.93 ± 0.21 ^a^	0
250 MPa, 5 min	5.79 ± 0.17 ^b^	0.48	4.02 ± 0.25 ^b^	1.91
350 MPa, 5 min	4.80 ± 0.30 ^c^	1.47	3.34 ± 0.12 ^c^	2.59
450 MPa, 5 min	<2.0	6.27	<2.0	5.93
550 MPa, 5 min	<2.0	6.27	<2.0	5.93

* Values are means ± standard deviation (*n* = 3). Means in every column with the different superscripts are significantly different (*p* < 0.05).

## Data Availability

Not applicable.
